# TOWARD A BETTER UNDERSTANDING OF SOCIAL EXCLUSION IN LATER LIFE

**DOI:** 10.1093/geroni/igad104.0221

**Published:** 2023-12-21

**Authors:** Marja Aartsen, Michal Myck

## Abstract

Social exclusion (SE) – or the separation of individuals and groups from mainstream society - is a serious problem with adverse health and well-being outcomes and increased societal costs. Older adults form a vulnerable group as they tend to be excluded for longer periods than younger people. A growing body of knowledge suggests that SE is multidimensional, including social, cultural, economic, material, and geographical aspects. There are however noticeable knowledge gaps, of which some are addressed in this symposium. Three presentations are based on large cross-national data sets, the final is a qualitative study on a rarely investigated but relevant social group, i.e., older Roma people. The first presenter shows how childless older adults in Europe feel more excluded than older adults with children and discusses how normative pressures can exclude people from society (data from the European Social Survey, wave 1 and 10). The second presenter explains how depopulation of geographical areas can act as driver of SE through material hardship, low social integration and lacking public infrastructure in the depopulated areas (data derived from SHARE, wave 4 and 6). Based on unique data from Poland and Germany, the third presenter shows how associations between markers of SE and well-being are modified by the macro-social context and characteristics of the individual. By using an intersectional life course perspective, the last presentation indicates how pathways in- and out SE are linked to social and societal processes and the political history of a country in a socially deprived group.

